# Case Report: Observation of early-onset high myopia with fundus tessellation changes in Coffin–Siris syndrome 9 (CSS9) and literature review

**DOI:** 10.3389/fped.2025.1603863

**Published:** 2025-08-26

**Authors:** Ruohao Wu, Yu Li, Zhanwen He, Zhe Meng, Wenting Tang, Liyang Liang

**Affiliations:** ^1^ Department of Children’s Neuroendocrinology, Sun Yat-sen Memorial Hospital, Sun Yat-sen University, Guangzhou, Guangdong, China; ^2^ Children’s Medical Center, Sun Yat-sen Memorial Hospital, Sun Yat-sen University, Guangzhou Guangdong, China; ^3^ Department of Research and Molecular Diagnostics, Sun Yat-sen University Cancer Center, Sun Yat-sen University, Guangzhou, Guangdong, China

**Keywords:** *SOX11*, Coffin-Siris syndrome 9, early-onset high myopia, fundus tessellation changes, ophthalmological phenotypic spectrum

## Abstract

Coffin-Siris syndrome 9 (CSS9), a rare congenital disorder caused by SRY-related HMG-box 11 gene (*SOX11*) deficiency, is characterized by high phenotypic heterogeneity including a wide spectrum of organ anomalies. Pathogenic variants in *SOX11* can induce ocular motor disorders and ocular deformities resulting in visual malfunctions. Here, we report a 10-year-old Chinese boy with early-onset high myopia (eoHM) and fundus tessellation changes with cone-rod cells dystrophy who presented with characteristic CSS phenotypes, including coarse facial features, neurodevelopmental disabilities, and fifth finger anomalies. By applying trio-based whole-exome sequencing, we identified a *de novo* variant in *SOX11*, NM_003108.4: c.1013 C>T, p. S338l, classified as likely pathogenic. A systematic literature review yielded 14 publications providing detailed data from 57 CSS9 cases. Quantitative analysis of the ophthalmological phenotypic spectrum of the 58 cases (including our proband) revealed that almost half (26/58, 44.83%) presented ophthalmological malformations; the most prevalent phenotype was ocular motor disorder (15/58, 29.31%); however, pathologic fundus change was only reported in our proband (1/58, 1.72%), suggesting that fundus examination may have been lacking in previous investigations of CSS9 cases. In summary, we report a CSS9 patient with eoHM and fundus tessellation changes, suggesting a potential role of *SOX11* in fundus oculi development. We recommend ophthalmological examination with fundus screening for individuals with CSS9 presenting with significant visual impairments, as ophthalmological malformation with extensive lesions is a potentially important feature of CSS9.

## Background

Coffin-Siris syndrome (CSS, OMIM#135900), a heterogeneous group of rare autosomal dominant syndromes, was first described and reported by Coffin and Siris in 1970 (1). Patients with CSS often present with global developmental delay/intellectual disability of varying severity, coarse facial features, typically including thick arched eyebrows, low-set anteriorly/posteriorly rotated ears, and facial hirsutism, and hypoplastic or absent fifth fingers or toes. These three phenotypes can be considered characteristic features of CSS and serve as clues for CSS diagnosis (2); however, affected individuals may also have heterogeneous malformations of various organs, presenting with high phenotypic variability (3, 4). Mutations in 12 different genes involved in subunits of the SWItch/Sucrose Nonfermentable (SWI/SNF) complex, a chromatin remodeling factor with an important role in cell fate determination and organ development, have been found to cause various CSS subtypes (5). Overall, the high heterogeneity of CSS has hindered full elucidation of the genotypic and phenotypic complexity of the condition and make clinical diagnosis of CSS particularly challenging. Hence, more descriptions of CSS cases are needed to enrich understanding of its phenotypic complexity.

Among CSS subtypes, CSS9 (OMIM#615866) is caused by heterozygous mutations in the SRY-related HMG-box 11 (*SOX11*) gene, which is located on chromosome 2p25.2 and is important in chromatin remodeling related to SWI/SNF complex functions (6). Recently, patients with CSS9 have been reported to exhibit fewer CSS-characteristic phenotypes, with a greater likelihood of multiple organ anomalies (7), including various types of ophthalmological abnormalities, such as ocular motor disturbances (8–11), glaucoma (8, 12), iris coloboma and cataracts (8, 9, 12). Ophthalmological abnormalities previously reported in individuals with CSS9 mainly present as deformities in external ocular structures, such as the eyelid, musculus oculi, and iris, but there are no reports of internal ocular structure malformations directly caused by *SOX11* mutation. Here, we report the case of a 10-year-old Chinese boy who carried a *de novo* missense *SOX11* variant and presented with some CSS-characteristic phenotypes, including coarse facial features, neurodevelopmental disorders, and fifth-finger abnormalities, as well as early-onset high myopia (eoHM) with typical fundus tessellation changes and cone-rod cells dystrophy. We also conducted a systematic literature review to explore the ophthalmological phenotypic spectrum of all reported CSS9 cases, providing new information to improve understanding of ophthalmological malformations in CSS9.

## Methods

### Patient and ethical compliance

A 10-year-old Chinese Han proband and his healthy parents were investigated in this study at the Children's Medical Center of Sun Yat-sen Memorial Hospital, Sun Yat-sen University. The proband's phenotypic data (including facial, limbic, and other dysmorphisms), cardinal results of auxiliary examinations, including auditory brainstem response tests, fundus examinations performed in our hospital, and outside medical records/reports, like measurements of intraocular pressure (IOP) and axial length (AL), optical coherence tomography (OCT) and electroretinography (ERG) tests, were collected during the proband's hospitalization in our department after informed written permission to use the proband's clinical data including individual outside medical records/reports, and unidentified facial and limbic phenotypes for publication was obtained from the proband's guardians. All procedures of the study were performed in accordance with the Declaration of Helsinki and approved by the Ethics Committee of Sun Yat-sen Memorial Hospital (Approval Number: SYSKY-2024-1121-01).

### Genetic investigation

Informed written consent for genetic testing was obtained from the proband's family before genetic investigation was performed. Routine genetic screening tests, including G-banded karyotyping, fragile X chromosome analysis, and chromosomal microarray analysis (CMA), were firstly performed on the proband according to standard protocols to exclude chromosomal aberrations, fragile X syndrome and potential pathogenic copy-number variants (CNVs). Next, genomic DNA was extracted from the peripheral blood of the proband and his parents via a commercialized peripheral blood genomic extraction kit (Qiagen, Shanghai, China) in accordance with the manufacturer's instructions. The purity and concentration of the obtained DNA were calculated via a NanoDrop 2000 (NanoDrop, Wilmington, USA) and a Qubit kit (Thermo Fisher Scientific, Waltham, USA) following the manufacturer's standard protocols. Finally, the extracted DNA from the proband and his parents was used for trio-based whole-exome sequencing (trio-WES).

In brief, we used an Illumina TruSeq exome kit (Illumina, San Diego, USA) to construct DNA libraries following the manufacturer's instructions. An Illumina NovaSeq 6000 (Illumina, San Diego, USA) was then used to conduct sequencing according to the standard protocols of the manufacturer. A total of∼10 GB of exome data/individual were obtained. Then, exome sequencing data analysis was conducted via a commercial pipeline developed by GeneRanger (Xunyin Biotech, Shanghai, China). Read alignment, indel region realignment, base quality recalibration, variant capture, and calling/transformation were performed via the Burrows–Wheeler aligner, Picard tool and Genome Analysis ToolKit. based on the standard criteria reported by the Genome Aggregation Database (gnomAD) (13). Variant filtering was set to a coverage depth > 10 with a minor allele frequency < 0.05%. The scoring and interpretation of the pathogenicity of the identified variants were based on the variant classification system developed by the American College of Medical Genetics (ACMG) (13). Validation of the trio-WES-detected variant was conducted via Sanger sequencing following standard protocols. The sequences of primers used for Sanger sequencing of the *SOX11* variant identified in this study were as follows: forward, CTACAACGTCGCCAAAGTGC; reverse, GTGCAGTAGTCGGGGAACTC. Protein modeling of the wild-type (SOX11*^WT^*) and mutant-type SOX11 (SOX11*^MT^*) proteins were modeled by AlphaFold tool (http://alphafold.ebi.ac.uk), and visualized by PyMol software (http://pymol.org/2/). The change of thermostability (measured via the change value of Gibbs free energy, *ΔΔ*G) in SOX11*^MT^* protein caused by the identified missense variant (p.S338l) was evaluated by mCSM tool (http://structure.bioc.cam.ac.uk/mcsm) (14).

### Literature review and analysis of the ophthalmological phenotypic spectrum

This systematic literature review aimed to obtain the genotypes and ophthalmological phenotypic spectra of CSS9 patients with pathogenic intragenic *SOX11* variants reported in peer-reviewed papers in the PubMed (https://www.ncbi.nlm.nih.gov/) and Online Mendelian Inheritance in Man (OMIM) databases (https://omim.org/). The literature review was conducted in compliance with the Preferred Reporting Items for Systematic Reviews and Meta-Analyses (PRISMA) statement (15). The PubMed database was comprehensively searched up to March 2025 via the MeSH terms “Coffin-Siris Syndrome” AND “SOX11 protein, human”. The OMIM database (http://omim.org/) was comprehensively searched up to March 2025 via the search term “SOX11”. The types of articles that met the eligibility criteria included case reports, case series, case‒control studies, and cohort studies. We also checked the reference parts of the already included publications and incorporated additional references containing CSS9 cases with full details of genotypes and phenotypes. We excluded experimental studies that explored the function or mechanism of *SOX11*. We also excluded general summary review papers and publications with insufficient phenotype and genotype information for the CSS9 patients.

After we obtained eligible papers, we collected data related to those reported CSS9 cases' genotypes and their respective ophthalmological phenotypes and quantitatively analyzed the incidence rates of different types of ophthalmological anomalies in those cases.

## Results

### Case presentation

A Chinese boy with neurodevelopmental disorders, comprising neurodevelopmental delay with autism spectrum disorder, and high myopia with hearing impairments, attended our children's medical center at 10 years old. He was the first child born to unrelated healthy parents via vaginal delivery following spontaneous labor at full-term. There were no pathological features or events during the fetal period. After birth, he was breast fed without feeding difficulties. Since infancy, his mother noticed that there were obvious delays in his language development and personal/social competence development, with sounds being repeated at 3 years of age and unmeaningful words being spoken at 5 years of age. He was diagnosed with autism spectrum disorder at 5 years old, based on autistic features, such as impairments in verbal and nonverbal communication and some repetitive patterns of behavior. The patient's mother consulted a clinic physician from the Department of Ophthalmology and Otorhinolaryngology elsewhere when he was 5 years old because of bilateral strabismus with right eyelid ptosis. On specialized physical examination, he was found to have myopia and astigmatism in both eyes and hearing impairment in both ears; however, his mother refused further hospitalization and examination. He experienced three bouts of febrile convulsions at the age of 8 years. All electroencephalograms (EEGs), including one urgent EEG, demonstrated no abnormal findings.

**Figure 1:**
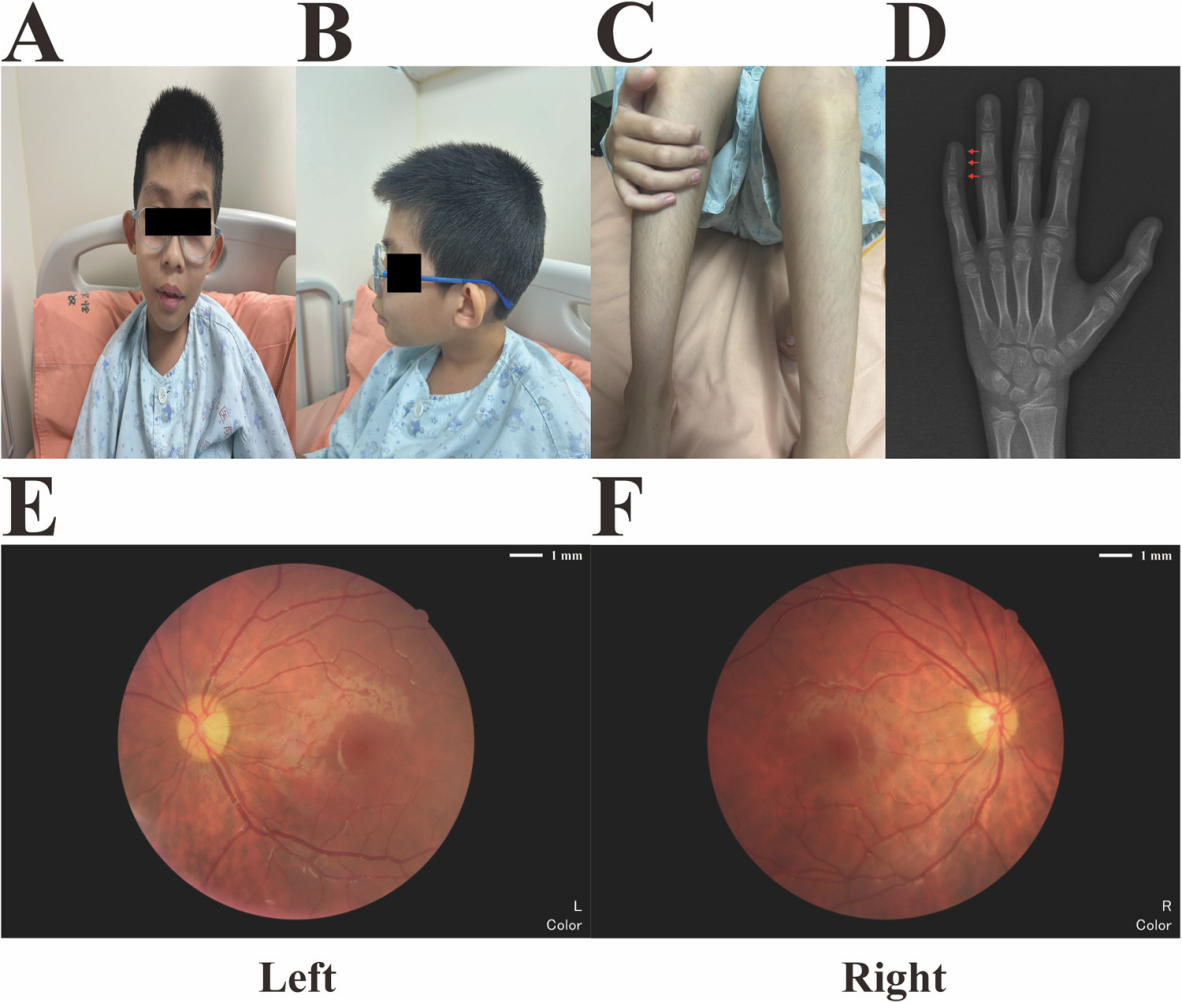
Main phenotypic characteristics of the proband. **(A)** Coarse facial features (thick and arched eyebrows with low-set anteriorly rotated ears) and bilateral strabismus with right eyelid ptosis. **(B)** Facial hirsutism and high myopia with thick-lens glasses. **(C)** Hypertrichosis of the proband's shins. **(D)** X-radiograph of the proband's left hand at the age of 10, which revealed distal phalanx hypoplasia of the fifth finger and mild clinodactyly (red arrows). **(E,F)** Fundus imaging revealed obvious fundus tessellation changes in both eyes of the proband.

During his hospitalization at our medical center aged 10 years, physical examination revealed coarse facial features, primarily thick, arched eyebrows, low-set, anteriorly rotated ears, and bilateral strabismus, with right eyelid ptosis. In addition, the patient presented with facial hirsutism and wore thick-lens glasses due to myopia and astigmatism (Figures 1A,B). Best corrected visual acuity (BCVA) values of the right (oculus dexter, OD) and left (oculus sinister, OS) eyes were 0.3 and 0.2, respectively, indicating high myopia. Due to financial constraints and inability of the proband to fully cooperate with comprehensive ophthalmic examinations, the proband's guardians declined repetition of identical ophthalmic investigations that had been conducted in another medical facility, including measurements of IOP and AL, as well as OCT and ERG tests. Thus, the results of those ophthalmic examinations were mainly based on the proband's medical records/reports. As demonstrated in Table 1, the IOP of both eyes were measured to be normal (OD, 14 mmHg; OS, 15 mmHg), while the AL of both eyes were measured to be 28.97 mm (OD) and 30.00 mm (OS), respectively. Macular OCT reports revealed no abnormalities in either eye, while ERG showed dystrophy of cone and rod cells in both eyes, evidenced by significantly decreased amplitudes of a- and b-waves in scotopic and photopic adaptations, which may explain the observed fundus changes in this male. Moreover, he exhibited hypertrichosis on his shins (Figure 1C), and distal phalanx hypoplasia of the fifth finger, mild clinodactyly in the left hand (Figure 1D). His weight, height, and head circumference were 21.3 kg (< −3.0 SD), 126.5 cm (< −3.0 SD), and 48 cm (< −2.0 SD, microcephaly), respectively, indicating that he had microcephaly and growth retardation. Auditory brainstem response test revealed bilateral cochlear damage, with a normal hearing threshold (60 dBnHL) (Supplementary File S1). Color images from fundus examination in our center revealed typical fundus tessellation changes in the proband's eyes (Figures 1E,F), indicating potential pathological myopia. All other laboratory and neuroimaging test results were unremarkable. There was no pertinent familial history of myopia or similar conditions and, considering the onset age of myopia (5 years) in this patient and his BCVA values (OD: 0.3, OS: 0.2) with typical fundus tessellation changes, a diagnosis of eoHM was made.

**Table 1:** Ophthalmological examination summary of the proband with CSS9 and eoHM based on his outside medical reports at the age of 10.

Eye	OD	OS
BCAV	0.3	0.2
IOP (mmHg)	14.0	15.0
AL (mm)	28.97	30.00
Results of macular OCT	Normal	Normal
Results of ERG	Scotopic adaptation: a-wave ↓ and b-wave ↓ (both were around 0 μV) Photopic adaptation: a-wave ↓ and b-wave ↓(both were around 0 μV)	Scotopic adaptation: a-wave ↓ and b-wave ↓ (both were around 0 μV) Photopic adaptation: a-wave ↓ and b-wave ↓ (both were around 0 μV)

OD, oculus dexter; OS, oculus sinister; BCAV, best corrected visual acuity; IOP, intraocular pressure; AL, axial length; OCT, optical coherence tomography; ERG, electroretinography.

### Genetic investigation results

**Figure 2:**
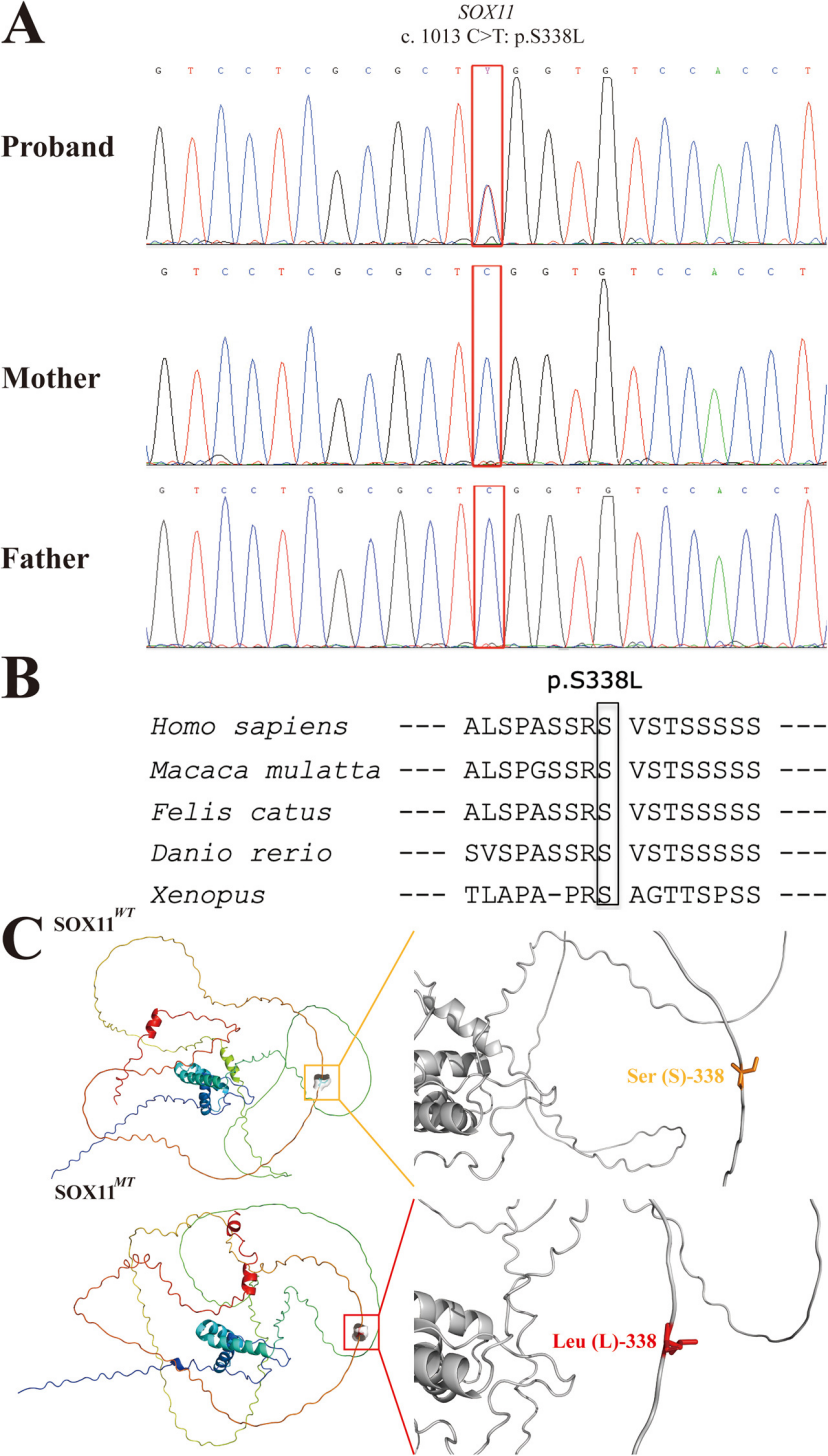
Sanger sequencing electropherograms and bioinformatic analysis of the identified *SOX11* variant (c.1013C>T:p.S338l). **(A)** Sanger sequencing electropherograms of the proband and his parents are shown. The mutational locus is indicated by a red box. **(B)** Comparative sequence alignments revealing that the missense variant (p. S338l) occurred at an evolutionarily conserved amino acid locus in the SOX11 protein (black box). **(C)** The 3D structure images of the SOX11*^WT^* (*upper*) and SOX11*^MT^* (*lower*) proteins and their corresponding zoomed images of Ser (S)-338 and Leu (L)-338 sites. The orange box indicates the original polar amino acid residue Ser (S), and the red box indicates the mutant nonpolar amino acid residue Leu (L). SOX11*^WT^*, wild-type SOX11; SOX11*^MT^*, mutant-type SOX11.

Considering the phenotypes of the proband, a congenital syndrome involving multiple organ malformations was considered the most plausible diagnosis. Trio-whole exome sequencing (WES) was conducted using samples from the proband and his parents after the results of routine genetic examinations of the proband, including G-band karyotype, CMA, and fragile X chromosome analysis, were negative. Trio-WES analysis revealed a heterozygous missense variant, NM_003108.4: c.1013C>T, p.S338l, in *SOX11* in the proband. We rated the pathogenicity of this *SOX11* variant according to the ACMG variant classification scoring rules (13). Sanger sequencing confirmed that neither parent carried the variant (Figure 2A), indicating that it was a *de novo* change (PS2). Moreover, the variant (rs1339382294) had a very low minor allele frequency of 0.000009949 in the gnomAD database (v4.1.0) and was absent from the East Asian population database (PM2_supporting). Missense variants in *SOX11* are the most common cause of CSS9 and few benign missense variants have been reported in this gene (8); thus, the missense variant in *SOX11* provides supporting evidence for pathogenicity (PP2). Additionally, amino acid conservation analysis revealed that S (serine, Ser) at site 338 in the SOX11 protein was highly evolutionarily conserved in *Homo sapiens*, *Macaca mulatta*, *Felis catus*, *Danio rerio*, and *Xenopus* (as indicated by the black box in Figure 2B). The structure of the SOX11*^WT^* and SOX11*^MT^* proteins were constructed via AlphaFold tool. Visualization of the 3D-structures via PyMol software revealed that the original polar amino acid residue Ser (S)-338 had been replaced with the mutant nonpolar amino acid residue Leu (L)-338 (Figure 2C). The results of mCSM prediction showed that the corresponding change value of Gibbs free energy (ΔΔG) in MT protein was negative (−0.418 kcal/mol), indicating that the identified missense variant (p.S338l) can cause impaired thermostability to SOX11 protein, leading to the instability of structure in SOX11*^MT^* protein (PP3). Finally, considering the evidence for variant pathogenicity alongside the CSS-related features of the proband, including coarse facial features and fifth finger anomalies (PP4), our data confirm that the c.1013C>T (p.S338l) *SOX11* variant can be scored as likely pathogenic (PS2 + PM2_supporting + PP2 + PP3 + PP4). Hence, this *SOX11* variant identified via trio-WES analysis is the most plausible causative variant in our proband, and a genetic diagnosis of CSS9 was made for the patient.

### Results of the literature review and ophthalmological phenotypic spectrum analysis

**Figure 3:**
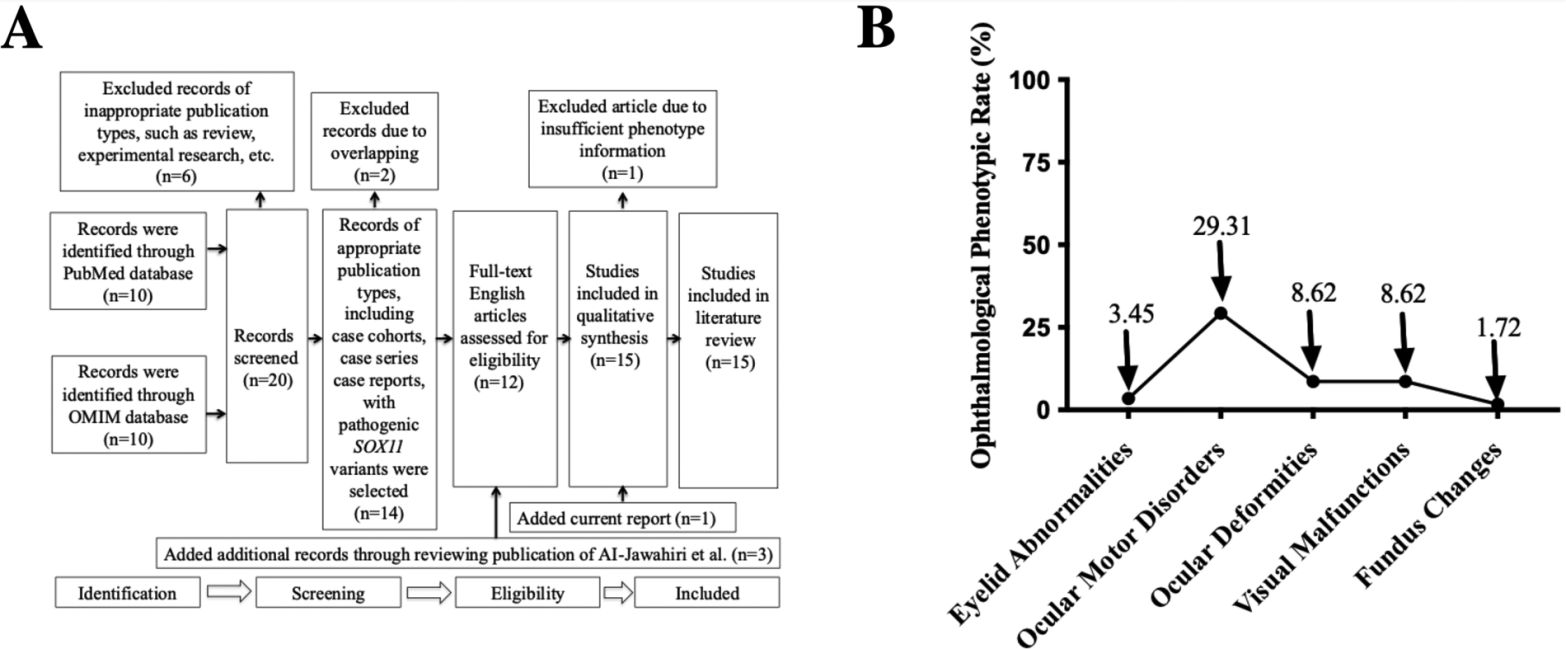
PRISMA flowchart and ophthalmological phenotypic spectrum analysis. **(A)** A flowchart showing systematic review results in the PubMed and OMIM databases for articles containing CSS9 patients carrying pathogenic *SOX11* variants. “*n*” indicates the number of included or excluded publications at different stages. **(B)** A distribution plot demonstrating the incidence of different ophthalmological phenotypes in all reported cases with pathogenic *SOX11* variants. PRISMA, Preferred Reporting Items for Systematic Reviews and Meta-Analyses; CSS9, Coffin-Siris Syndrome 9.

As shown in Figure 3A, we conducted a systematic literature search based on the statement of PRIMSA (15) in the PubMed and OMIM databases, and included our current report; finally, a total of 14 publications with our report that met the eligibility criteria (3, 4, 6–12, 16–20) were obtained. After excluding patients with CNVs that included other adjacent genes, 58 patients with CSS9 carrying pathogenic or likely pathogenic single nucleotide variants in *SOX11* were selected for further review of detailed information related to their genotypes and ophthalmological phenotypes and phenotypic spectrum analysis.

Comprehensive and careful review of the phenotypic spectrum of ophthalmological malformations among published CSS9 cases, alongside our current case, demonstrated that almost half (26/58, 44.83%) exhibited various types of ophthalmological anomalies, including eyelid abnormalities, ocular motor disorders, ocular deformities, visual malfunctions, and fundus changes, suggesting that ophthalmological malformations may be an important common feature phenotype in patients with CSS9. Detailed information about the ophthalmological phenotypes of these 58 patients is summarized in Supplementary File S2. As shown in Figure 3B, among the different types of ophthalmological phenotype reported, the most prevalent were ocular motor disorders, with an incidence rate of 29.31% (15/58), followed by ocular deformities (5/58, 8.62%) and visual malfunctions (5/58, 8.62%). However, fundus changes were only reported in our patient, with an incidence rate of 1.72% (1/58).

## Discussion

The rapid development and popularization of trio-WES technology has resulted in more frequent discovery of genetic causes in individuals with unexplained syndromes involving multiple organ malformations, including CSS9 (7, 21–23). Since 2014, when Tsurusaki et al. used WES technology to identify pathogenic mutations in *SOX11* in two unrelated children with CSS-featured phenotypes, including hypertrichosis, impaired intellectual development, and hypoplastic distal fingers/toes (6), more than 50 individuals worldwide with multiple organ malformations carrying *SOX11* variants have been reported and the phenotypic spectrum of CSS9 has expanded annually; hence, new reports describing CSS9-related phenotypes can help identify further genotype‒phenotype correlations related to this congenital disorder.

Here, we describe a Chinese Han boy with a *de novo* missense variant in *SOX11* (NM_003108.4: c.1013C > T, p.S338l). His phenotypes were consistent with various previously recorded clinical manifestations in patients with CSS9, including neurodevelopment impairments, coarse facial features, and fifth finger malformation. Scoring of the pathogenicity of the identified variant according to ACMG guidelines (13) led to a diagnosis of CSS9 in this patient. The *SOX11* variant identified in this report is located outside the HMG-box domain, which is a primary pathogenic hotspot domain in cases of CSS9 (24). Nevertheless, AI-Jawahiri et al. also reported a patient who died at the neonatal stage and harbored a *SOX11* missense variant outside of the HMG-box domain, which caused significant impaired transactivating activity (8), supporting the deleterious effect of the *SOX11* missense variant outside the HMG-box detected in our study; however, the difference in pathogenicity between variants within and outside the HMG domain requires further exploration through in-depth mechanistic experiments.

The patient in this study presented eoHM with fundus tessellation changes. Meanwhile, he also showed AL elongation but the macular OCT results showed no abnormalities in both eyes. Meanwhile, the ERG findings revealed dystrophy of cone-rod cells in his eyes, which indicated that the observed fundus changes may be largely associated with a syndrome-specific retinal dystrophy in this patient. However, we should note that a single macular OCT scan at age 10 does not guarantee lifelong absence of macular pathology. Therefore, we cannot exclude the potential secondary effects on the retinal phenotype caused by high myopia with the AL elongation, long-term follow up of this patient is still required. EoHM, a subtype of high myopia with onset before school age, is a pathogenic form of myopia caused mainly by genetic factors (25), and has been detected in systemic disorders and congenital syndromes (called “syndromic eoHM”), such as Donnai-Barrow syndrome (26) and Rubinstein-Taybi syndrome 2 (27). In current report, considering the syndromic conditions of this male, he can also be diagnosed with syndromic eoHM. Actually, EoHM has already been previously documented in CSS; Huang X et al. reported one CSS1 male exhibiting eoHM and carrying an *ARID1B* frameshift variant in 2024 (28). Using a STRING dataset (https://cn.string-db.org) and performing protein‒protein interaction (PPI) analysis, they predicted that the ARID1B/ARID1A protein may cause the eoHM phenotype via interactions with several high myopia-related proteins, including FGFR3, SOX4, ERBB3, and ASXL1. Using the same bioinformatic methods, we uploaded SOX11 and 10 high myopia-related proteins (ERBB3, FGFR3, SOX4, COL9A1, COL2A1, COL11A1, ASXL1, COL18A1, P3H2 and P4HA2) to STRING database and generated a PPI network (Supplementary File S3), revealing that SOX11 has multiple interactions, including co-expression and experimentally determined interactions, with three high myopia-related proteins (COL9A1, COL2A1, and COL11A1), as well as protein sequence homology with another high myopia-related protein, SOX4. Therefore, we propose that SOX11 may directly or indirectly affect proteins with strong links to high myopia, such as COL9A1, COL2A1, and COL11A1, by affecting the expression or function of SOX4, ultimately causing the clinical phenotype of eoHM and dystrophy of cone-rod cells in CSS9 individuals. Further, Nunomura et al. revealed that heterozygous deficiency of *Sox11* (*Sox11*^+/−^) may cause failure of embryonic eye development, including eyelid closure in a mouse model, by affecting fibroblast growth factor (FGF10)-mediated signaling (29). Moreover, Wurm et al. reported that homozygous *Sox11* deficiency (*Sox11*^−/−^) can cause ocular anterior segment dysgenesis in a mouse model and that lack of bone morphogenetic protein 7 (BMP7) signaling may causatively contribute to ophthalmological phenotypes in *Sox11*^−/−^ mice (30). These findings could explain the observed ophthalmological anomalies, such as eyelid ptosis and ocular motor dysfunction, in our proband; however, it remains unclear how *SOX11* dysfunction influences fundus oculi development, especially the dystrophy of cone-rod cells. This warrants further study through the identification of more cases and functional experiments, including validation of the abovementioned bioinformatics-predicted signaling, which will be the focus of our future work.

By systematically reviewing published literature and analyzing the ophthalmological phenotypic spectrum of 58 reported CSS9 cases (including our current case), we demonstrated that 44.83% (26/58) of individuals with CSS9 had various types of ophthalmological malformations or symptoms. This finding is consistent with a report of symptoms in patients with CSS1 (48.6%, 69/143) (31), suggesting that the ophthalmological phenotype can be considered a common feature in patients with CSS9, rather than an occasional phenotype, as suggested by Diel et al. (12). Thus, more medical attention focused on clinical assessment of ocular symptoms in children with CSS9 is warranted. Further, among the various ophthalmological phenotypes detected in patients with CSS9, we found that the most prevalent was ocular motor disorders (29.31%, 15/58), reinforcing the conclusion of Hanker et al. that ocular motor dysfunction could be an important diagnostic clue for CSS9 (9). In contrast, fundus changes were only discovered in our patient among those with CSS9 reported, representing an incidence rate of 1.72% (1/58); we speculate that this phenotype may have been largely overlooked in previous investigations of CSS9 cases, and suggest that extensive ophthalmological tests, including fundus screening, should be conducted routinely for individuals with CSS9, particularly those with significant visual impairment, as extensive ophthalmological malformations involving multiple lesions may occur in patients carrying *SOX11* variants.

Our current study is limited in that we were unable to exclude the possibility that there have been previous cases of CSS9 with fundus tessellation changes, as fundus examination has largely been ignored in many prior studies; this may have led to unavoidable ascertainment bias, and the novelty of our CSS9 case with fundus tessellation changes should be interpreted with considerable caution.

## Conclusions

In conclusion, we applied trio-WES technology to identify a likely pathogenic *SOX11* variant in a Chinese boy exhibiting multiple ophthalmological malformations, including eyelid ptosis, strabismus and eoHM with fundus tessellation changes and dystrophy of cone-rod cells, indicating that SOX11 has a potential role in fundus oculi development. Moreover, by systematically reviewing the ophthalmological phenotypic spectrum of reported CSS9 cases, we suggested that CSS9 children showing visual impairments should be aware of potential risks of having multiple ophthalmological lesions and need extensive ophthalmological screening, including fundus examination.

## Data Availability

The datasets presented in this study can be found in online repositories. The names of the repository/repositories and accession number(s) can be found below: https://ngdc.cncb.ac.cn/search/all?&q=GVM000927, GVM000927.
