# Supplementary material for: Case Report: Observation of early-onset high myopia with fundus tessellation changes in Coffin–Siris syndrome 9 (CSS9) and literature review
Source: Front Pediatr. 2025 Aug 26;13:1603863. doi: 10.3389/fped.2025.1603863 (PMC12417530; doi:10.3389/fped.2025.1603863)
Supplement: Supplementary file 1 [file Datasheet1.pdf]

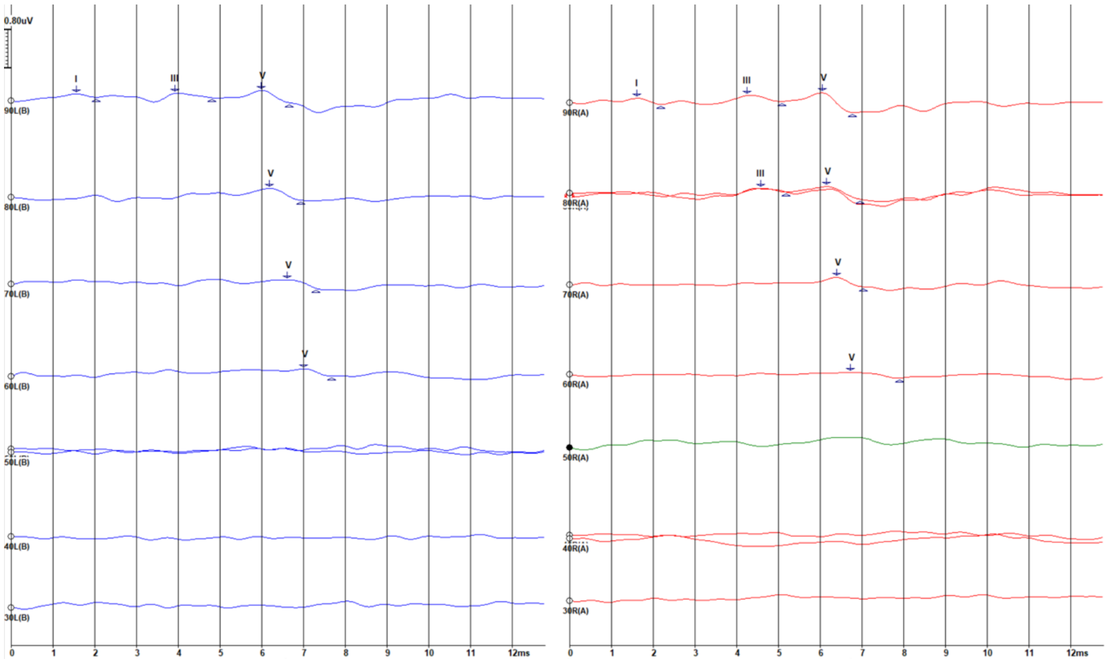

Supplementary File 1 Routine audiometric test of the proband. Auditory brainstem response testing revealed bilateral cochlear damage with a normal hearing threshold (60 dBnHL). Blue line: left ear; red line: right ear.
