# Supplementary material for: Case Report: Observation of early-onset high myopia with fundus tessellation changes in Coffin–Siris syndrome 9 (CSS9) and literature review
Source: Front Pediatr. 2025 Aug 26;13:1603863. doi: 10.3389/fped.2025.1603863 (PMC12417530; doi:10.3389/fped.2025.1603863)
Supplement: Supplementary file 3 [file Datasheet3.pdf]

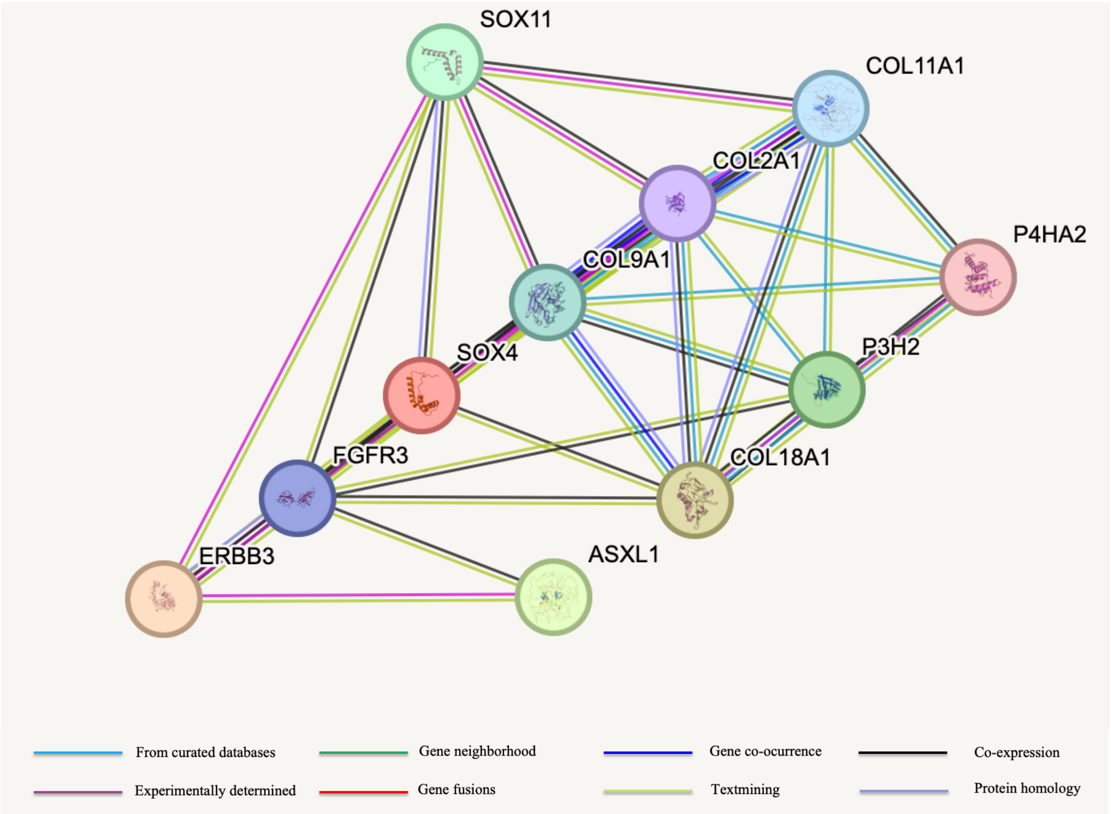

Supplementary File 3 Diagram of protein-protein interactions. The results of protein-protein interaction analysis in the STRING online database revealing multiple interactions among SOX11 protein and 10 high myopia-related proteins (ERBB3, FGFR3, SOX4,COL9A1, COL2A1, COL11A1, ASXL1, COL18A1, P3H2 and P4HA2).
